# Targeted ZnO@CuEA Nanoplatform for Cuproptosis‐Based Synergistic Cancer Therapy

**DOI:** 10.1111/jcmm.70636

**Published:** 2025-06-02

**Authors:** Hao Zhang, Guoyan Liu

**Affiliations:** ^1^ Department of Gastrointestinal Surgery, Xiang'an Hospital of Xiamen University, School of Medicine Xiamen University Xiamen China

**Keywords:** cellular senescence, cuproptosis, ferroptosis, ZnO@CuEA

## Abstract

Gastric cancer is a common malignant tumour. Copper‐induced cell death, a recently discovered form of metal ion‐related cell death, has garnered significant attention from researchers. We synthesised a multifunctional nanoparticle, ZnO@CuEA, and characterised its morphology using transmission electron microscopy. Cytotoxicity was analysed via CCK8 assays and calcein‐AM staining. The tumour‐targeting capability of ZnO@CuEA was validated using confocal microscopy and in vivo imaging experiments. RNA‐seq and proteomic analysis were conducted to assess changes in mRNA and protein expression before and after ZnO@CuEA treatment. Lysosomal β‐galactosidase staining was employed for cellular senescence detection, and protein expression levels were analysed via Western blot. Finally, in vivo experiments demonstrated the tumour‐inhibitory effect of ZnO@CuEA. ZnO@CuEA is a multifunctional nanoparticle capable of targeting tumour cells and inducing cuproptosis. In vivo experiments showed that ZnO@CuEA exhibits significant antitumor activity. The multifunctional nanoparticles synthesised in this study provide a novel therapeutic approach for cancer treatment.

**Abbreviations**
Cu: Copper
CuE: Cu (II)‐Elesclomol
GC: Gastric cancer
TEM: transmission electron microscopy
XRD: X‐ray diffraction

## 1 Introduction

Gastric cancer (GC) is a common malignant tumour. The diet, smoking, *Helicobacter pylori* infection, and inflammation contribute to GC risk [1]. GC, despite the availability of various therapeutic strategies, including chemotherapy, surgery, radiotherapy, targeted therapies, and immunotherapy, the prognosis for GC patients remains unsatisfactory. Therefore, identifying alternative and effective therapeutic approaches for GC is of critical importance.

Copper (Cu), an essential trace element for maintaining normal physiological functions in the human body, is mainly derived from dietary intake [2]. It is critically involved in numerous cellular physiological and pathological activities [3, 4, 5]. An imbalance in copper homeostasis has been linked to the development of various diseases. Scientists have found that delivering Cu ions into tumour cells to raise intracellular copper concentrations can induce cell death. In 2022, Tsvetkov et al. coined the term “cuproptosis” to describe this copper‐triggered form of cell death. Their research indicated that cuproptosis is characterised by the accumulation of lipoylated dihydrolipoamide S‐acetyltransferase and the loss of Fe‐S cluster proteins [6]. The buildup of Cu within mitochondria ultimately leads to proteotoxic stress and subsequent cell death.

Currently, oligonucleotides are widely used in various applications due to their advantages, such as low synthesis cost, abundant target candidates, and high specificity and selectivity. They can serve as primers for DNA synthesis, gene probes, and more. AS1411 aptamer is a synthetic G‐rich DNA aptamer that targets nucleolin. In certain tumours, such as colorectal cancer, breast cancer, GC, and lung cancer, nucleolin is significantly expressed on the tumour cell membrane [7, 8, 9, 10]. AS1411 is often used to conjugate nanoparticles for precise drug delivery to tumour cells, reducing the nonspecific cytotoxicity of chemotherapy drugs on normal cells.

Nanoparticles are widely applied in the treatment of diseases. Research has shown that polyoxometalates with a nanocapsule‐like structure containing copper can achieve controlled copper ion release when exposed to ionising radiation, thereby boosting the localised anti‐tumour efficacy of radiotherapy and overcoming acquired resistance to it [11]. In another study, elesclomol and copper were co‐loaded into nanoparticles using a polymer sensitive to reactive oxygen species, resulting in a formulation that not only exerts cytotoxic effects on cancer cells but also stimulates an anti‐tumour immune response [12]. Additionally, a self‐amplified cuproptosis nanoparticle utilising celastrol can amplify cuproptosis and activate immunogenic cell death [13].

In this study, we synthesised ZnO@CuEA, a nanoparticle capable of targeting tumour cells and inhibiting tumour progression. ZnO@CuEA exerts antitumor effects through multiple mechanisms, including cuproptosis, ferroptosis, cellular senescence, and activation of the P53 signalling pathway. These findings offer new insights into cancer therapy.

## 2 Materials and Methods

### 2.1 Synthesis of Cu (II)‐Elesclomol (CuE)

Elesclomol (100 mg) was purchased from MedChemExpress (Monmouth Junction, USA) was dissolved in 25 mL of DMSO, and mixed thoroughly by stirring. Subsequently, 150 mg of CuCl_2_ (Aladdin, Shanghai, China) was added, followed by the addition of 250 mL of deionised water. The mixture was stirred at room temperature for 15 min, then collected and dialyzed overnight using a dialysis bag (MWCO: 3500 Da).

### 2.2 Synthesis of ZnO@CuEA

To prepare ZnO@CuEA, 200 mL of the CuE solution described above was combined with 30 μL of 100 μM AS1411 (MedChemExpress, Monmouth Junction, USA) and 117 mg of ZnO (Aladdin, Shanghai, China). The mixture was stirred overnight in the dark at room temperature.

### 2.3 Characterisation

Using transmission electron microscopy (TEM) (FEI, Netherlands) to observe the surface morphology of nanoparticles. Elemental analysis was conducted via X‐ray diffraction (XRD) (Xtalab Synergy, Netherlands). The hydrodynamic size and zeta potential were detected using DLS (Zetasizer Nano ZS90, UK).

### 2.4 Cell Culture

The MFC, GSE‐1, and MKN45 were cultured at 37°C in a 5% CO_2_ incubator. All media were supplemented with 10% fetal bovine serum (Gibco, USA), 50 μg/mL penicillin, and 50 μg/mL streptomycin (Gibco, USA). Cell line MKN45 was maintained in 1640 medium (Gibco, USA).

### 2.5 Cytotoxicity Assay

The CCK‐8 assay (MCE, Shanghai, China) was employed to evaluate cell viability under varying nanoparticle concentrations. After 24 h of treatment, 10 μL of CCK‐8 reagent was added, and the absorbance at 450 nm was subsequently measured. To assess hemolytic activity, fresh mouse blood was used, with PBS and distilled water serving as negative and positive controls, respectively.

### 2.6 Flow Cytometry

Cells were first digested with trypsin lacking EDTA, followed by centrifugation at 300 g for 5 min at 4°C. After discarding the supernatant, the pellet was washed twice with PBS and resuspended in 100 μL Binding Buffer to yield a single‐cell suspension. PI and Annexin V‐FITC staining solutions were added, and the mixture was incubated in the dark at room temperature for 10 min. Subsequently, 400 μL Binding Buffer was added, and the samples were gently mixed prior to analysis.

### 2.7 Western Blot

Following a 24‐h nanoparticle treatment, total proteins were extracted using RIPA buffer. Protein samples were resolved via 10% SDS‐PAGE and transferred onto PVDF membranes. After blocking with 5% non‐fat milk for 1.5 h at room temperature, membranes were incubated with primary antibodies at 4°C overnight and with HRP‐conjugated secondary antibodies for 1–2 h. Signal detection was performed using ECL reagents.

### 2.8 RNA‐Seq and Bioinformatics Analyses

Isolating total RNA from cultured cells using TRIzol reagent (Takara, China). Subsequent RNA sequencing and bioinformatics analyses were conducted by Shanghai Sangon Biotech.

### 2.9 ELISA Experiment

After 24‐h cell treatments, culture supernatants were collected and centrifuged at 1000 g for 20 min. The resulting supernatants were processed according to the instructions provided in the ELISA kit. Optical density at 450 nm was measured, and protein concentrations were calculated accordingly.

### 2.10 siRNA Interference

Custom‐designed siRNAs targeting GPX4 and FDX1 (Table S1), as well as a negative control siRNA, were synthesised by Genepharma. Cells were transfected with these siRNAs using Lipofectamine 3000 (Thermo Fisher) as per the manufacturer's protocol and incubated for 48 h.

### 2.11 Determination of Intracellular ROS

Cells were evenly seeded into 6‐well plates and allocated into six groups. Upon reaching ~75% confluence, fresh medium containing different nanoparticles was added. After 2 h of incubation, the medium was removed, and 1 mL of pre‐diluted ROS detection solution (Beyotime, Shanghai) was added to each well. Following a 20‐min incubation at 37°C, cells were washed with PBS. Fluorescence intensity reflecting ROS levels was then observed under a fluorescence microscope.

### 2.12 Quantitative Real‐Time PCR

Extracting total RNA from cultured cells using TRIzol reagent (Takara, China). cDNA synthesis was performed using the PrimeScript RT Kit (Takara), and quantitative PCR was carried out with SYBR Premix EX Taq (Takara). Calculating relative gene expression levels by the 2^−ΔΔCT^ method. In Table S1, primer sequences are listed.

### 2.13 LC–MS/MS

MKN45 cells were assigned to either the control group or the ZnO@CuEA treatment group, with three replicates per group. Protein extraction from cells was performed using a 1% sodium deoxycholate solution. Protein samples were analysed and quantified using LC–MS/MS on a Thermo Fisher Scientific system.

### 2.14 Cellular Senescence Detection

The culture medium was removed, and cells were washed once with PBS. Then, β‐galactosidase staining fixative was added at room temperature for 15 min. The fixative was removed, and the cells were washed three times with PBS. Next, 1 mL of β‐galactosidase staining solution (Beyotime, Shanghai, China) was added, and the cells were incubated overnight at 37°C. Cellular senescence was observed under a standard optical microscope.

### 2.15 Animal Experiments

All animal experiments were approved by the Ethics Committee of Xiamen University. BALB/c mice were randomly divided into groups and injected subcutaneously with MFC cells. Treatments were administered on the day of cell implantation, and tumour growth was monitored every 2 days for 2 weeks.

### 2.16 Statistical Analysis

Data were analysed using *t*‐tests for two‐group comparisons and one‐way ANOVA for multiple groups. Wilcoxon and Kruskal‐Wallis tests were used for non‐normally distributed data. Statistical significance was defined as *p* < 0.05 (*p* < 0.01 for highly significant).

## 3 Results and Discussion

### 3.1 Synthesis and Characterisation of ZnO@CuEA

**FIGURE 1:**
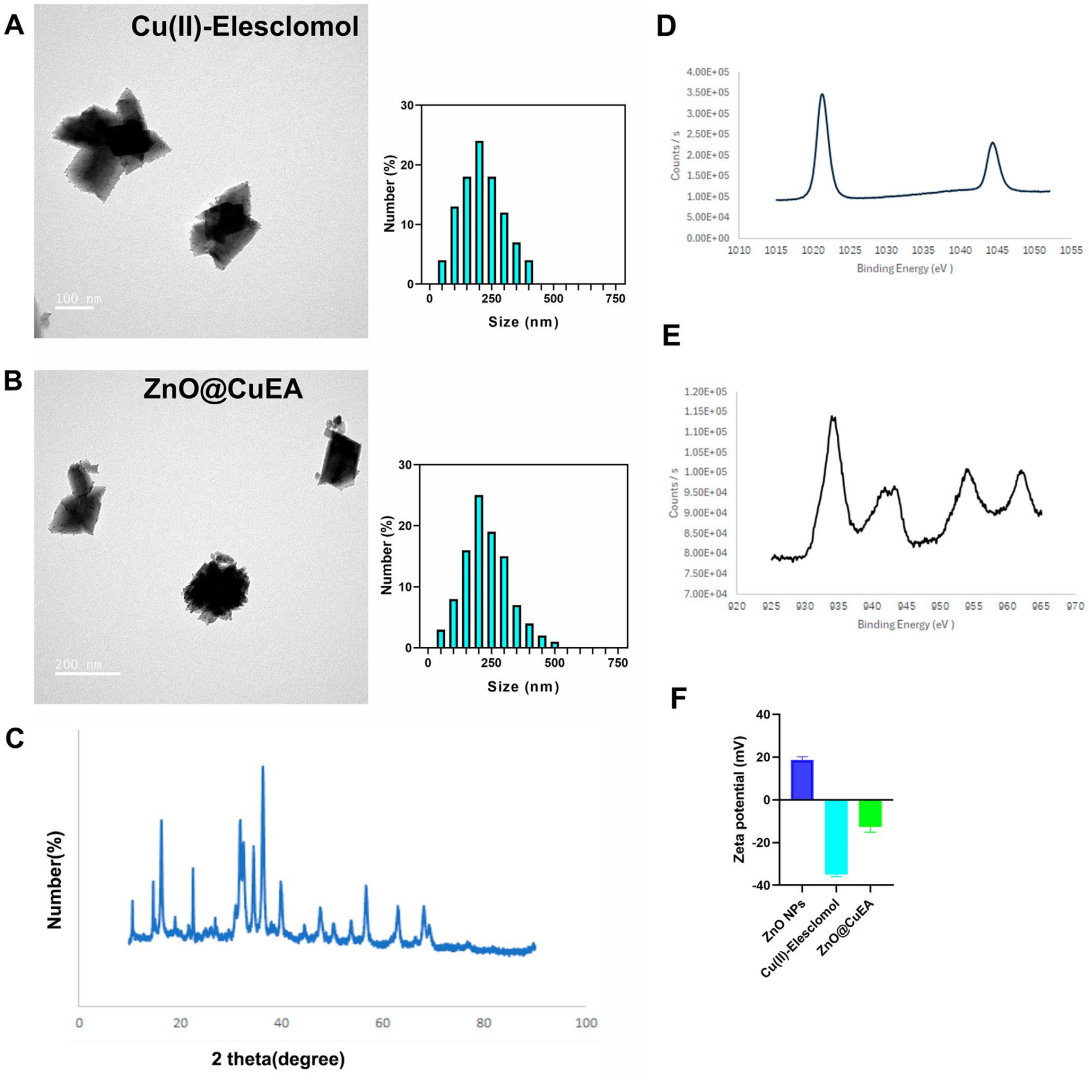
Characterisation of nanoparticles. (A) TEM images and particle size distribution of CuE. (B) TEM images and particle size distribution of ZnO@CuEA. (C) XRD analysis of ZnO@CuEA. (D, E) XPS spectra of Cu2p and Zn2p in ZnO@CuEA. (F) The Zeta potentials of ZnO NPs, CuE and ZnO@CuEA NPs in aqueous solution.

First, the CuE was synthesised. TEM results showed that the diameter of CuE particles ranged from 150 to 250 (Figure 1A). After adding ZnO and AS1411, TEM analysis revealed that the synthesised ZnO@CuEA nanoparticles also had a particle size distribution of 150–250 nm (Figure 1B). XRD analysis of ZnO@CuEA showed dominant peaks at 2θ = 16.3°, 22.5°, 31.8°, 34.5° and 36.4° (Figure 1C). The X‐ray photoelectron spectroscopy results indicate that the Cu2p peak is at 934.18 eV, while the Zn2p peak is at 1021.28 eV (Figure 1D,E). Additionally, we measured the zeta potential of ZnO@CuEA (Figure 1F). ZnO NPs exhibit a positive potential, whereas CuE and ZnO@CuEA exhibit negative potentials.

### 3.2 Tumour Suppression by ZnO@CuEA

**FIGURE 2:**
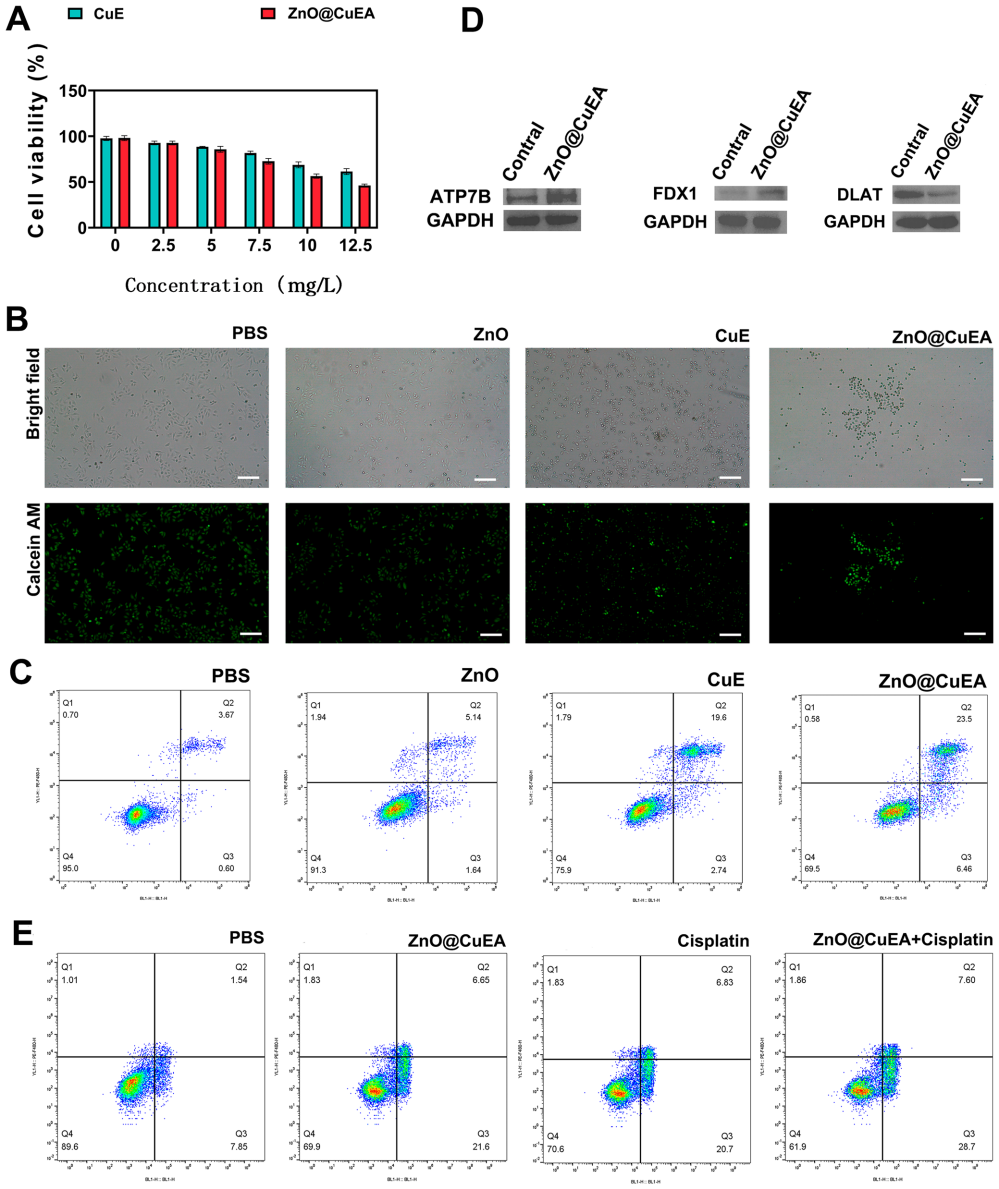
Cytotoxicity and copper‐induced cuproptosis of nanoparticles. (A) Tumour cell cytotoxicity of CuE and ZnO@CuEA assessed by the CCK‐8 assay. (B) Cytotoxicity verified by calcein‐AM staining. (C) Apoptosis induction by nanoparticles detected via flow cytometry. (D) Changes in ATP7B, FDX1 and DLAT expression before and after nanoparticle treatment. (E) Synergistic killing effect of ZnO@CuEA and cisplatin (100 μM) on GC cells.

The cytotoxic effects of CuE and ZnO@CuEA on MKN45 cells were examined by setting a concentration gradient (Figure 2A). Calcein‐AM staining experiments confirmed the cytotoxicity of ZnO, CuE, and ZnO@CuEA, with ZnO@CuEA exhibiting the strongest tumour toxicity (Figure 2B). ZnO@CuEA significantly promoted apoptosis in MKN45 cells (the cell viability of the ZnO@CuEA group was 67.5 ± 2.29 compared with 94.67 ± 1.53 in the control group; *p* < 0.05; *n* = 3) (Figure 2C).

To investigate the cuproptosis‐inducing effect of ZnO@CuEA, the expression levels of ATP7B, FDX1, and DLAT proteins were measured. ZnO@CuEA was found to significantly upregulate the expression of both ATP7B and FDX1 (Figure 2D). However, DLAT was significantly downregulated under the effect of ZnO@CuEA. The ATP7B transporter can mediate cellular copper efflux as needed, maintaining a relatively stable intracellular copper ion concentration [14]. When excessive copper ions enter tumour cells and trigger cell death, FDX1 is subsequently downregulated [15]. Copper metabolism disorders caused by FDX1 deficiency are closely associated with poor overall survival and prognosis in certain cancer patients, such as those with colon or liver cancer. In tumour cells, excess Cu^2+^ can directly bind to DLAT, blocking the mitochondrial tricarboxylic acid cycle, leading to the accumulation of acylated DLAT and inducing cuproptosis [16]. These three genes play crucial roles in the process of cuproptosis.

Whether ZnO@CuEA can enhance current GC treatment is an important question. Cisplatin is a commonly used drug for treating GC patients, but significant drug resistance occurs during cisplatin therapy, highlighting the importance of developing combination therapies. Cisplatin exerts its antitumor effects by interfering with signal transduction and cell regulatory mechanisms, such as the activation of ERK, phosphorylation of p53, upregulation of p21, GADD45, Mdm2, and the phosphorylation of AKT at ser136 of the Bcl‐2‐related death promoter, leading to cell cycle arrest and triggering apoptosis [17]. ZnO@CuEA mainly induces cell death through the cuproptosis pathway, which mechanistically cooperates with cisplatin's anti‐tumour action. Therefore, we compared the therapeutic synergy between ZnO@CuEA and cisplatin. Flow cytometry analysis showed that, compared with ZnO@CuEA (the cell viability was 70.13 ± 0.78; *n* = 3) or cisplatin alone (the cell viability was 71.22 ± 1.21; *n* = 3), the combined treatment (the cell viability of the combined treatment group was 62.06 ± 2.02 compared with 90.92 ± 1.35 in the control group; *p* < 0.05; *n* = 3) significantly induced apoptosis in GC cells (Figure 2E).

### 3.3 Targeting Effects of ZnO@CuEA

**FIGURE 3:**
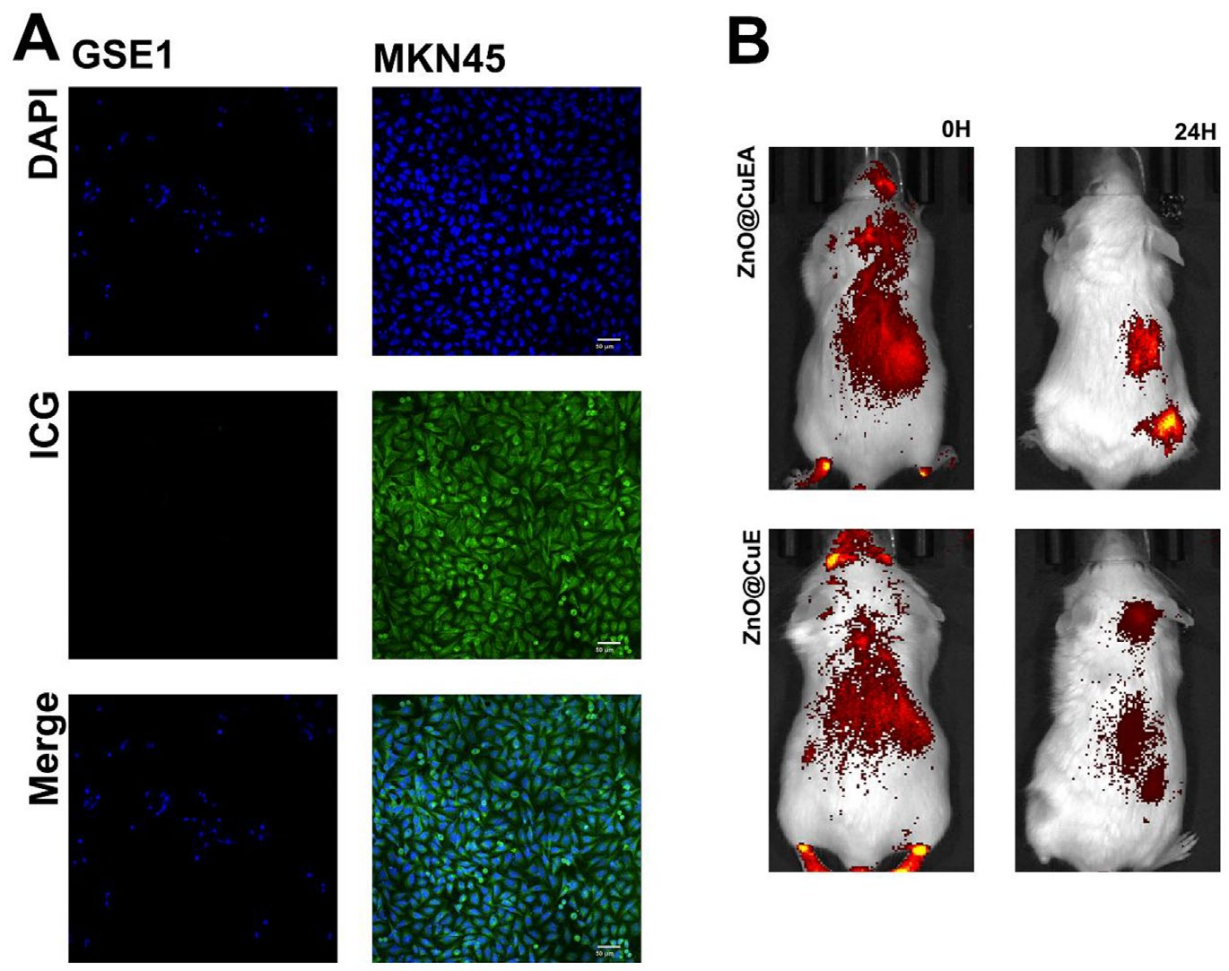
Targeting effects of ZnO@CuEA. (A) Targeting effects of nanoparticles on tumour cells and normal cells. (B) Targeting effects of nanoparticles on tumour tissues in mice.

Aptamers are essentially short single‐stranded DNA or RNA molecules that typically fold into three‐dimensional structures and bind to target molecules with high affinity and specificity. Due to their high specificity in nanobiotechnology, aptamers are widely used for the screening, enrichment, and isolation of target cells [18, 19, 20]. AS1411 is known to have targeting properties for tumour cells where nucleolin is overexpressed [21]. After incubating ZnO@CuEA containing ICG with gastric epithelial cells and GC cells for 2 h, confocal microscopy revealed strong accumulation of ZnO@CuEA in MKN45 cells (Figure 3A), while weaker accumulation was observed in GSE‐1 cells. In vivo, subcutaneous MFC tumour models were established in Balb/c mice. Fluorescence nanoparticles containing ICG were injected via the tail vein (5 mg/kg), and localization was observed at 0 h and 24 h. Nanoparticles with AS1411 showed significantly stronger fluorescence intensity in tumour tissues compared to those without AS1411, which were rapidly metabolised after 24 h (Figure 3B). These results demonstrated the excellent tumour‐targeting ability of ZnO@CuEA in both in vitro and in vivo experiments.

### 3.4 Transcriptomic Analysis

**FIGURE 4:**
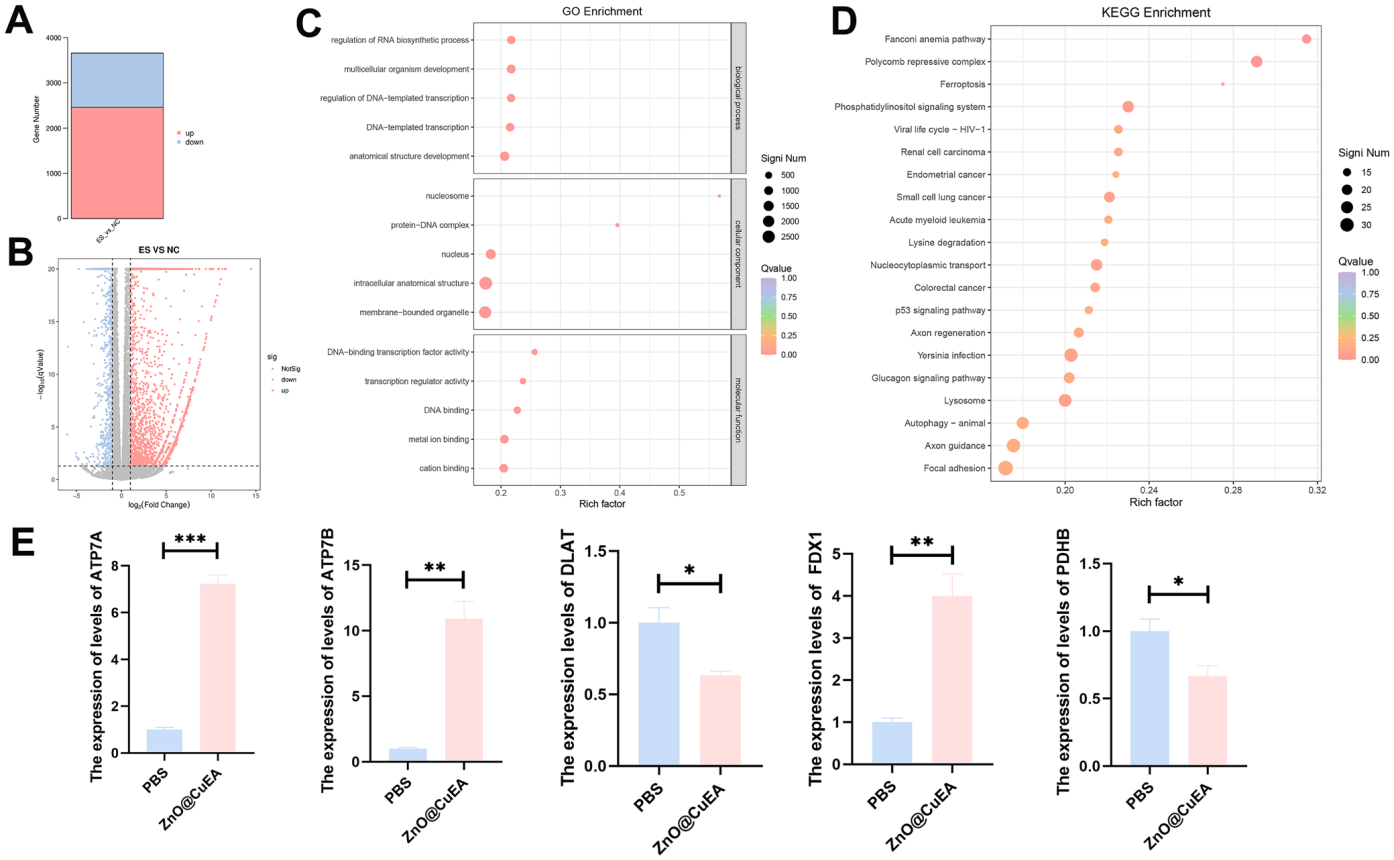
Transcriptomic analysis and verification. (A) Number of differentially expressed genes from transcriptomic analysis. (B) Volcano plot of differentially expressed genes. (C, D) GO and KEGG functional enrichment analysis. (E) mRNA expression levels of key cuproptosis‐related genes. *p < 0.05, **p < 0.01, and ****p < 0.0001.

Transcriptome sequencing was performed on MKN45 cells treated with ZnO@CuEA. A total of 3659 differentially expressed genes were identified, including 2461 upregulated genes and 1198 downregulated genes (|log FC| > 1, FDR < 0.05) (Figure 4A,B). Functional enrichment analysis of these genes revealed that in GO enrichment, ZnO@CuEA mainly regulated nuclear functions, such as the regulation of DNA‐templated transcription and DNA‐binding transcription factor activity (Figure 4C). In KEGG enrichment analysis, significantly enriched pathways included ferroptosis, p53 signalling pathway, and autophagy (Figure 4D).

Next, several cuproptosis‐related genes were selected, and their expression levels were verified by qPCR. The results showed that FDX1, ATP7B and ATP7A were upregulated in ZnO@CuEA‐treated MKN45 cells, whereas DLAT and PDHB were significantly downregulated after nanoparticle treatment (Figure 4E). PDHB is located in the mitochondria and is an enzyme that catalyses the conversion of glucose‐derived pyruvate into acetyl‐CoA, playing a vital role in oxidative phosphorylation [22].

### 3.5 Proteomics Analysis

**FIGURE 5:**
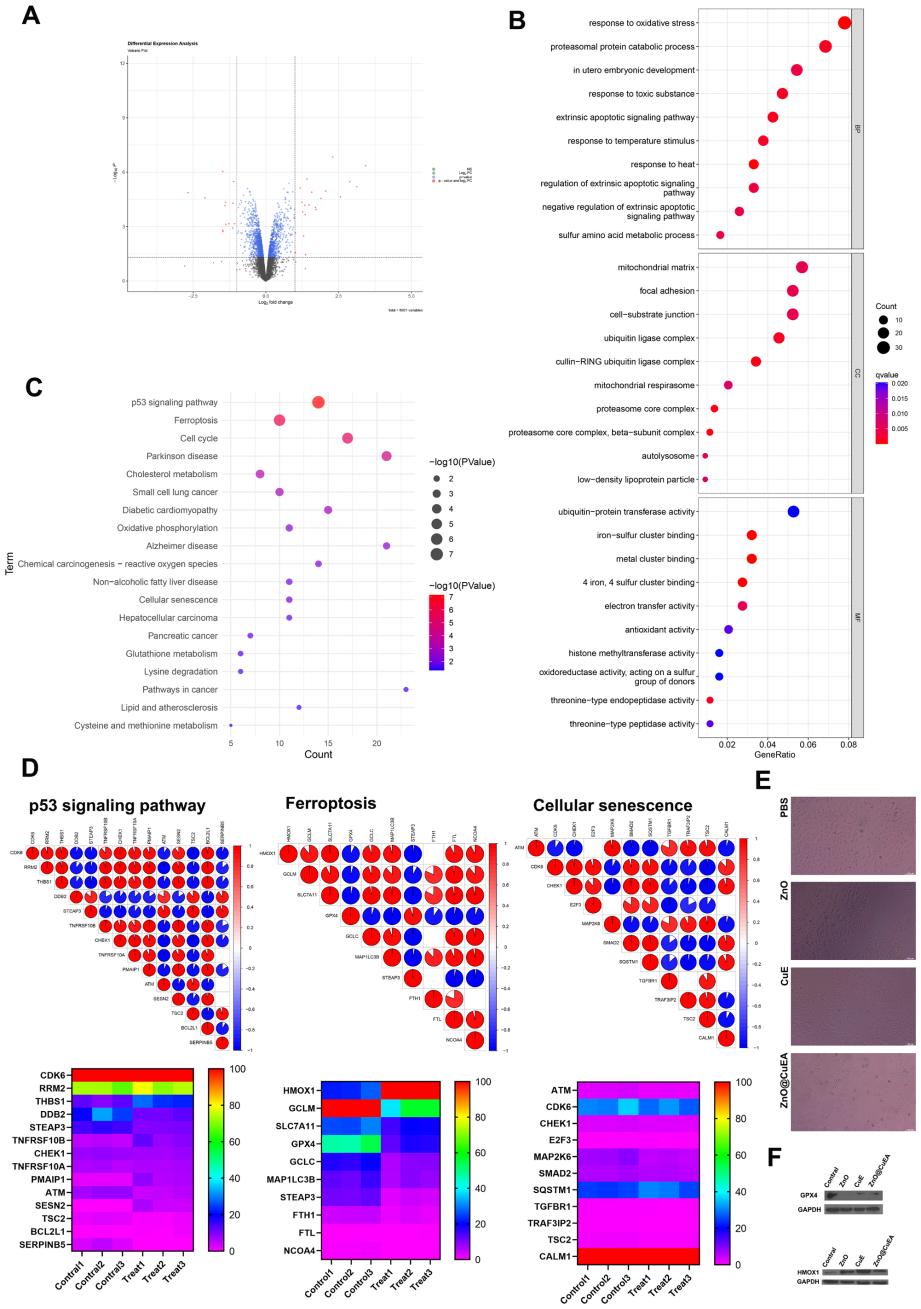
Differential protein analysis. (A) Differential protein analysis of MKN45 cells before and after ZnO@CuEA treatment. (B) GO enrichment analysis. (C) KEGG pathway analysis. (D) Gene expression and correlation analysis of relevant pathways. (E) Senescence staining assay. (F) Expression analysis of ferroptosis‐related genes.

Proteomic analysis of ZnO@CuEA‐treated MKN45 cells identified 458 differentially expressed genes (Figure 5A). KEGG and GO enrichment analyses revealed several interesting pathways, including the P53, ferroptosis, and cellular senescence pathways (Figure 5B,C). Heatmaps and correlation of gene expression in these pathways were visualised (Figure 5D). ZnO@CuEA was found to decompose into ZnO and CuE in cells under lysosomal action, with ZnO and CuE exerting distinct effects. Previous studies have suggested that Mn‐ZnO significantly accelerates the accumulation of lipid peroxides by promoting ROS generation and GSH depletion, leading to the inactivation of GPX4 and thereby inducing ferroptosis [23]. Researchers have designed a near‐infrared‐driven ROS self‐supplying nanomotor, in which ZnO can slowly release hydrogen peroxide into the acidic tumour microenvironment, providing sufficient components for the Fenton reaction required for ferroptosis [24]. The Zn^2+^ and H_2_O_2_ released by Mn‐ZnO NPs induce ubiquitination‐mediated proteasomal degradation of Mutp53, while the released Mn^2+^ and increased ROS levels activate the ATM‐p53‐Bax pathway, elevating the levels of WT P53 [25]. After treatment with zinc oxide nanoparticles, the p53 pathway in skin fibroblasts is activated, while the cell count decreases [26]. Our proteomics data also indicated that ZnO@CuEA induces cellular senescence. Further analysis revealed that CuE was the primary component responsible for cellular senescence induced by ZnO@CuEA (Figure 5E). Protein expression validation in the ferroptosis pathway showed a significant decrease in GPX4 and an increase in HMOX2 in the ZnO@CuEA group (Figure 5F).

### 3.6 Molecular Pathway Analysis of ZnO@CuEA

**FIGURE 6:**
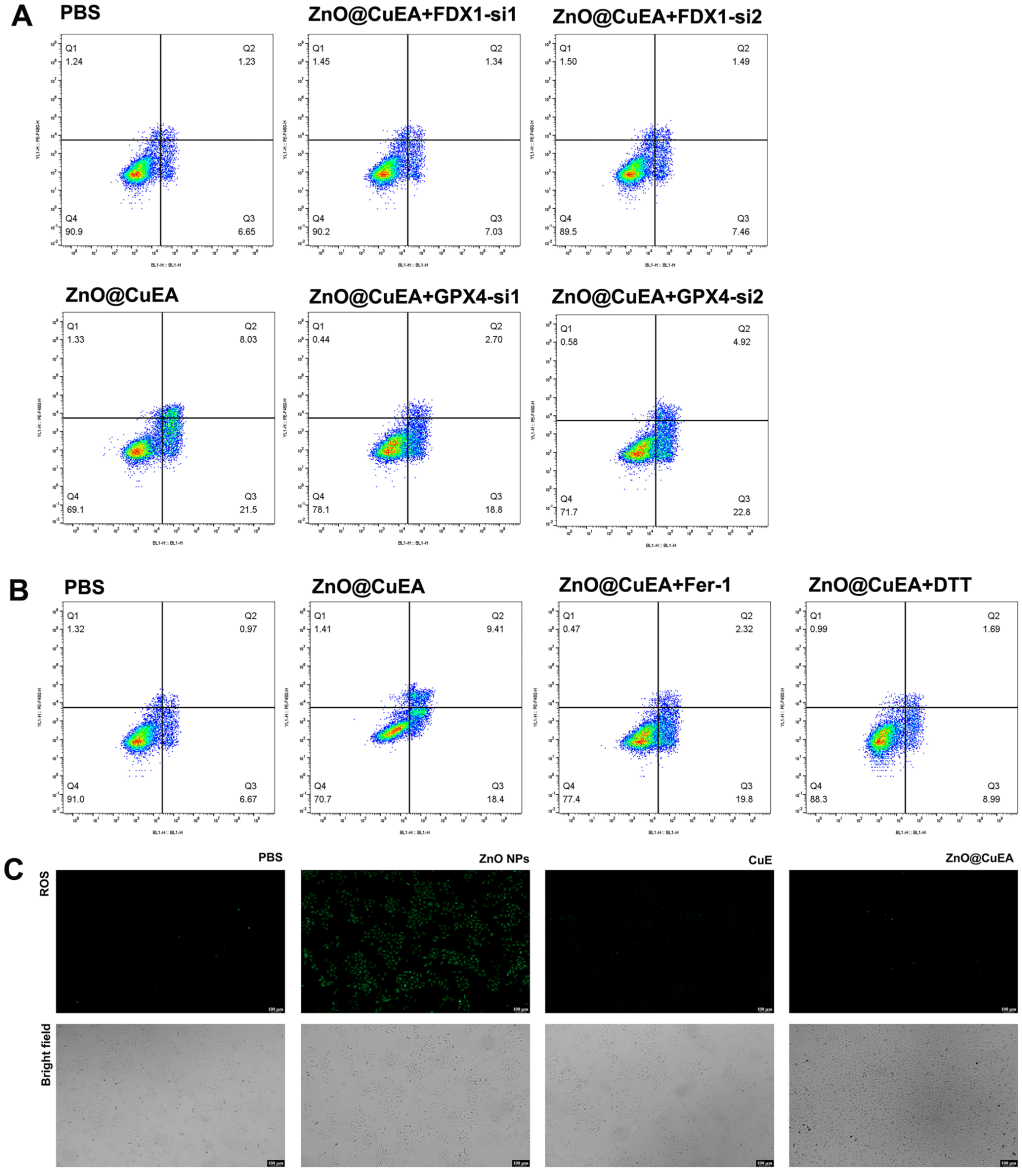
Molecular pathway analysis of ZnO@CuEA. (A) Resistance of tumour cells to ZnO@CuEA‐induced apoptosis after knockdown of FDX1 and GPX4. (B) Resistance of tumour cells to ZnO@CuEA‐induced apoptosis after the addition of Fer‐1 (4 μM) and DTT (60 μM). (C) Detection of ROS levels.

Transcriptomic and proteomic analyses revealed that ZnO@CuEA‐treated cells were enriched in ferroptosis‐related pathways, suggesting the need to distinguish more clearly between cuproptosis and ferroptosis mechanisms. We knocked down FDX1 and GPX4 separately and evaluated the apoptosis levels of MKN45 cells after ZnO@CuEA treatment. In cells with FDX1 knockdown, ZnO@CuEA‐induced apoptosis was significantly suppressed compared to ZnO@CuEA treatment alone (the cell viability of the ZnO@CuEA + FDX1 si1 and si2 group were 90.12 ± 1.58 and 90.55 ± 1.39 compared with 70.62 ± 1.45 in the ZnO@CuEA group; *p* < 0.05; *n* = 3). Knockdown of GPX4 also partially suppressed apoptosis, but the effect was less significant than that seen with FDX1 knockdown (Figure 6A).

We then added the ferroptosis inhibitor ferrostatin‐1 and the cuproptosis inhibitor DTT after ZnO@CuEA treatment and assessed apoptosis across the groups. Compared with ZnO@CuEA alone, the addition of DTT significantly inhibited ZnO@CuEA‐induced apoptosis (the cell viability of the ZnO@CuEA + DTT group was 87.06 ± 1.98 compared with 70.22 ± 1.45 in the ZnO@CuEA group; *p* < 0.05; *n* = 3), while ferrostatin‐1 only partially inhibited apoptosis (78.4 ± 0.79). Notably, the inhibitory effect of DTT was much stronger than that of ferrostatin‐1 (Figure 6B).

We also measured ROS levels in GC cells treated with ZnO, CuE, and ZnO@CuEA. ROS levels were significantly elevated in cells treated with ZnO nanoparticles, whereas the ROS increase in ZnO@CuEA‐treated cells was not significant, further suggesting that ZnO@CuEA does not primarily function via the ferroptosis pathway (Figure 6C).

### 3.7 Antitumor Effects of ZnO@CuEA In Vivo

**FIGURE 7:**
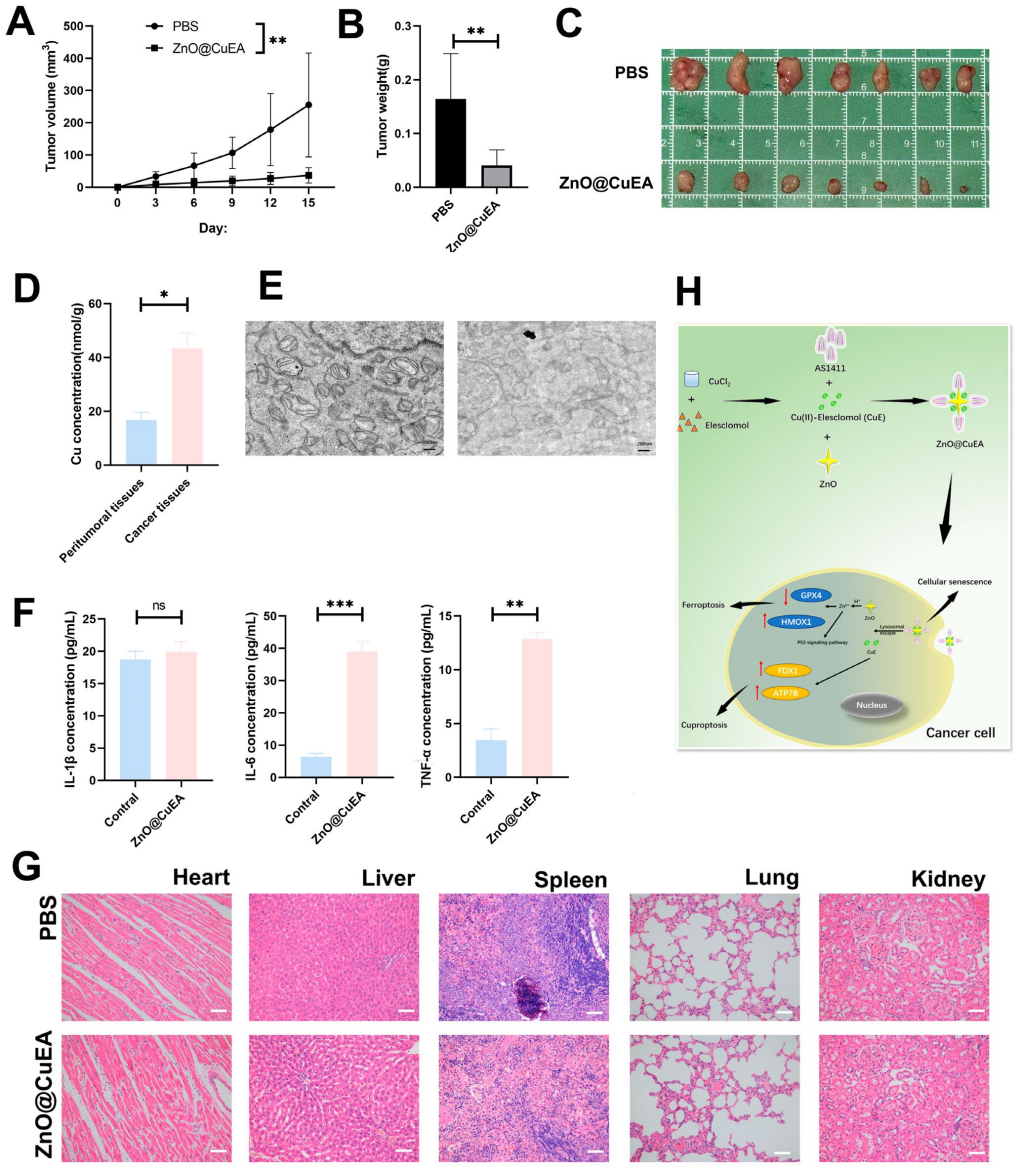
Validation of in vivo experiments. (A) Monitoring of tumour volume growth. (B) Tumour weight measurement. (C) Tumour morphology observation. (D) Detection of copper ion concentrations in tumour and adjacent tissues. (E) Intracellular localization of nanoparticles observed via TEM. (F) Detection of inflammatory cytokines in peripheral blood serum. (G) HE staining results of various organs in mice. (H) Schematic diagram of the synthesis process of ZnO@CuEA NPs and the mechanism. *p < 0.05, **p < 0.01, and ****p < 0.0001.

Subcutaneous tumour models were established in Balb/c mice, and when tumour volumes reached 150 mm^3^, mice were randomly divided into two groups: a ZnO@CuEA group and a control group. Tumour volume was recorded every 3 days, and tumour mass was measured at the end of the experiment. ZnO@CuEA significantly suppressed tumour progression (Figure 7A–C). To further verify and ensure the selective accumulation and distribution of ZnO@CuEA in tumour tissues, we measured Cu ion concentrations in both tumour and adjacent non‐tumour tissues. The results showed that the copper ion concentration in tumour tissues was significantly higher than that in adjacent tissues (Figure 7D). Additionally, we performed TEM to observe the intracellular localization of ZnO@CuEA. We found that ZnO@CuEA was internalised by cells and exerted its effects in the cytoplasm, with TEM images revealing significant morphological changes in mitochondria after cellular uptake of ZnO@CuEA (Figure 7E). Biocompatibility studies are essential for the application of nanoparticles. We measured pro‐inflammatory cytokines in the peripheral blood serum of mice, including TNF‐α, IL‐6, and IL‐1β. The results showed significant increases in TNF‐α and IL‐6, while IL‐1β levels remained relatively unchanged (Figure 7F). Studies have shown that serum levels of IL‐1β, IL‐6, and TNF‐α are significantly increased in copper‐exposed mouse models [27]. Other studies have found that in tumour cells, 5‐FU can increase the levels of TNF‐α and IL‐1β, but ZnO nanocomposites loaded with 5‐fluorouracil can reduce these levels [28]. This suggests that ZnO nanoparticles may play an important role in reducing IL‐1β concentrations in the peripheral blood of mice. HE staining of major organs showed no evident toxic effects or structural changes, indicating that ZnO@CuEA exhibits low biotoxicity while effectively inhibiting tumour growth (Figure 7G).

## 4 Conclusions

In this study, novel ZnO@CuEA nanoparticles were successfully developed, exhibiting excellent biocompatibility and tumour‐suppressing effects. ZnO@CuEA NPs were shown to inhibit tumour progression through cuproptosis (Figure 7H). We anticipate that our findings will promote the development of multifunctional nanomaterials and facilitate the clinical application of copper‐ and zinc‐based tumour inhibitors in biomedical therapy and research.

## Author Contributions

**Hao Zhang:** conceptualization (lead), data curation (lead), formal analysis (lead), investigation (lead), methodology (lead), project administration (lead), resources (lead), software (lead), validation (lead), visualization (lead), writing – original draft (lead), writing – review and editing (lead). **Guoyan Liu:** funding acquisition (lead), supervision (lead).

## Ethics Statement

The authors have nothing to report.

## Consent

The authors have nothing to report.

## Conflicts of Interest

The authors declare no conflicts of interest.

## Supporting information


Table S1

## Data Availability

The data that support the findings of this study are available from the corresponding author upon reasonable request.
