# Supplementary material for: Targeted ZnO@CuEA Nanoplatform for Cuproptosis‐Based Synergistic Cancer Therapy
Source: J Cell Mol Med. 2025 Jun 2;29(11):e70636. doi: 10.1111/jcmm.70636 (PMC12128471; doi:10.1111/jcmm.70636)
Supplement: Supplementary file 1 — Table S1 [file JCMM-29-e70636-s001.docx]

Supplementary Table 1. Sequence of oligonucleotides used in this study.

| Names | Sequence (5’-3’) |
| --- | --- |
| FDX1 siRNA1 | GGUGAAACAUUAACAACCA |
| FDX1 siRNA2 | ATCCCCTCCAGCATCCATCT |
| GPX4 siRNA1 | CAGGGAGUAACGAAGAGAU |
| GPX4 siRNA2 | GTGGATGAAGATCCAACCCAA |
| ATP7A forward primer | GCGCCAGATTCTTCCAGGAT |
| ATP7A reverse primer | ACCAGCCTCCGAAAAACTGTA |
| ATP7B forward primer | TGGAGATGGCGCACAAGATAA |
| ATP7B reverse primer | CACAGCCAGAACCTTCCTGA |
| DLAT forward primer | GCCACTGTTGGATTTGAGAGC |
| DLAT reverse primer | TCAATATCCTCAGGCTTGCCA |
| FDX1 forward primer | CAGCGGCCTGCTGAGG |
| FDX1 reverse primer | ACTGTTATTTTATCTTCTGAGCTGC |
| PDHB forward primer | CTGCGCTGCAGGTGACA |
| PDHB reverse primer | TTTCTTCCACAGCCCTCGAC |
| GPX4 forward primer | GTGGATGAAGATCCAACCC |
| GPX4 reverse primer | TTGTCGATGAGGAACTTGG |
| GAPDH forward primer | GAGTCAACGGATTTGGTCGT |
| GAPDH reverse primer | GACAAGCTTCCCGTTCTCAG |
